# Accelerated Iron Corrosion by Microbial Consortia Enriched from Slime-like Precipitates from a Corroded Metal Apparatus Deployed in a Deep-sea Hydrothermal System

**DOI:** 10.1264/jsme2.ME23089

**Published:** 2024-06-06

**Authors:** Satoshi Wakai, Sanae Sakai, Tatsuo Nozaki, Masayuki Watanabe, Ken Takai

**Affiliations:** 1 Institute for Extra-cutting-edge Science and Technology Avant-garde Research (X-star), Japan Agency for Marine-Earth Science and Technology (JAMSTEC), Yokosuka, Japan; 2 PRESTO, Japan Science and Technology Agency (JST), Tokyo, Japan; 3 Submarine Resources Research Center, Research Institute for Marine Resources Utilization, Japan Agency for Marine-Earth Science and Technology (JAMSTEC), Yokosuka, Japan; 4 Frontier Research Center for Energy and Resources, School of Engineering, The University of Tokyo, Japan; 5 Department of Planetology, Graduate School of Science, Kobe University, Kobe, Japan

**Keywords:** microbiologically influenced corrosion, deep sea, microbial community, methanogen

## Abstract

Microbiologically influenced corrosion refers to the corrosion of metal materials caused or promoted by microorganisms. Although some novel iron-corrosive microorganisms have been discovered in various manmade and natural freshwater and seawater environments, microbiologically influenced corrosion in the deep sea has not been investigated in detail. In the present study, we collected slime-like precipitates composed of corrosion products and microbial communities from a geochemical reactor set on an artificial hydrothermal vent for 14.5 months, and conducted culture-dependent and -independent microbial community ana­lyses with corrosive activity measurements. After enrichment cultivation at 37, 50, and 70°C with zero-valent iron particles, some of the microbial consortia showed accelerated iron dissolution, which was approximately 10- to 50-fold higher than that of the abiotic control. In a comparative ana­lysis based on the corrosion acceleration ratio and amplicon sequencing of the 16S rRNA gene, three types of corrosion were estimated: the methanogen-induced type, methanogen–sulfate-reducing bacteria cooperative type, and sulfate-reducing *Firmicutes*-induced type. The methanogen-induced and methanogen–sulfate-reducing bacteria cooperative types were observed at 50°C, while the sulfate-reducing *Firmicutes*-induced type was noted at 37°C. The present results suggest the microbial components associated with microbiologically influenced corrosion in deep-sea hydrothermal systems, providing important insights for the development of future deep-sea resources with metal infrastructures.

Metal corrosion phenomena cause significant economic losses, including the deterioration of existing infrastructures (Cámara *et al.*, 2022; Xu *et al.*, 2023). Microorganisms are involved in these phenomena, which are referred to as microbiologically influenced corrosion (MIC). Characteristic forms of corrosion have been observed in MIC, and localized corrosion significantly affects the service lifespan of metal materials. In addition to the corrosion form, abnormally fast corrosion rates and sudden occurrence are also problematic. When rapid and sudden localized corrosion occurs in pipelines, secondary environmental pollution results from leaked oil (Jacobson, 2007), leading to significant social issues.

Although research on MIC has a long history (Garett, 1891; Gaines, 1910), limited information is available on the causal microorganisms and their mechanisms. Various corrosive microorganisms with high corrosion activity have been identified in recent years, and in addition to sulfate-reducing bacteria (SRB) that have attracted attention for a long time, iron-corrosive methanogens, iron-corrosive nitrate-reducing bacteria, and iron-corrosive acetic acid-producing bacteria have been isolated (Dinh *et al.*, 2004; Mori *et al.*, 2010; Uchiyama *et al.*, 2010; McBeth *et al.*, 2011; Wakai *et al.*, 2014; Kato *et al.*, 2015; Iino *et al.*, 2015; Hirano *et al.*, 2022). The corrosion mechanism of these microorganisms comprises two types: chemical MIC (CMIC), which is indirectly affected by microbial metabolites, such as acids, corrosive gases, and oxidants, and electrical MIC (EMIC), which is affected by the electrochemical activity of microorganisms (Enning and Garrelfs, 2014). The corrosion mechanism of SRB and methanogens with high corrosion activity corresponds to EMIC, which is enhanced by extracellular electron transfer (EET) ability (Deng *et al.*, 2015; Tsurumaru *et al.*, 2018; Liang *et al.*, 2021). Although the corrosion mechanism of these microorganisms has gradually been revealed, the detection of corrosive microorganisms in suspected cases of MIC in the field is challenging.

MIC occurs in various settings, including soil, freshwater, and seawater environments, and the degree of corrosiveness of MIC depends on a combination of metal materials, environmental factors, and microorganisms. In recent years, we have conducted long-term immersion tests in a freshwater environment and demonstrated that the microbial community structure markedly changes during the corrosion process of carbon steel and other steels, resulting in the accumulation of SRB (Wakai *et al.*, 2022a). Similarly, the eventual accumulation of SRB with the progression of corrosion has been reported not only in freshwater, but also in seawater environments (Ramírez *et al.*, 2016; Vigneron *et al.*, 2016; Li *et al.*, 2017). On the other hand, in the corrosion products of chromium content and martensitic stainless steel corroded in the same freshwater environment, the presence of SRB was negligible, while sulfur-oxidizing bacteria and electrochemically active microbes were clearly detected (Wakai *et al.*, 2022a, 2022b). Therefore, the composition and transition of microbial components associated with MIC are affected not only by environmental conditions and microbial sources, but also by the target metal materials; therefore, corrosion reproduction tests are important for understanding MIC in practical environments.

Although previous studies examined MIC in various terrestrial and marine environments, limited information is available on corrosion in deep-sea environments (Ma *et al.*, 2020; Melchers, 2021; Rajala *et al.*, 2022). Although deep-sea environments are far from human activities, a large amount of metal equipment has been installed for purposes such as submarine cable internet connections, seafloor resource development, and various forms of monitoring. Current knowledge of MIC may not be fully applicable to deep-sea environments with low or high temperatures under high hydrostatic pressure. A microbiome ana­lysis of the corrosion of deep-sea carbon steel over the course of 10 years indicated that sulfur-metabolizing microorganisms contribute to corrosion (Rajala *et al.*, 2022). Although these findings form the basis for understanding MIC in cold deep-sea environments, the microbial populations and mechanisms of MIC in deep-sea hydrothermal systems remain unknown.

Therefore, we herein examined high-temperature MIC in a deep-sea hydrothermal system. We collected slime-like precipitates from a corroded metal apparatus deployed in an artificial deep-sea hydrothermal vent, which were then cultivated with zero-valent iron medium under moderately thermal conditions. Using enriched microorganisms, we conducted comparative ana­lyses of corrosion abilities and microbial communities. The results obtained revealed accelerated corrosion by moderately thermophilic microorganisms and possible corrosion-associated components.

## Materials and Methods

### Operation of the Kuroko-ore cultivation apparatus

**Fig. 1.**
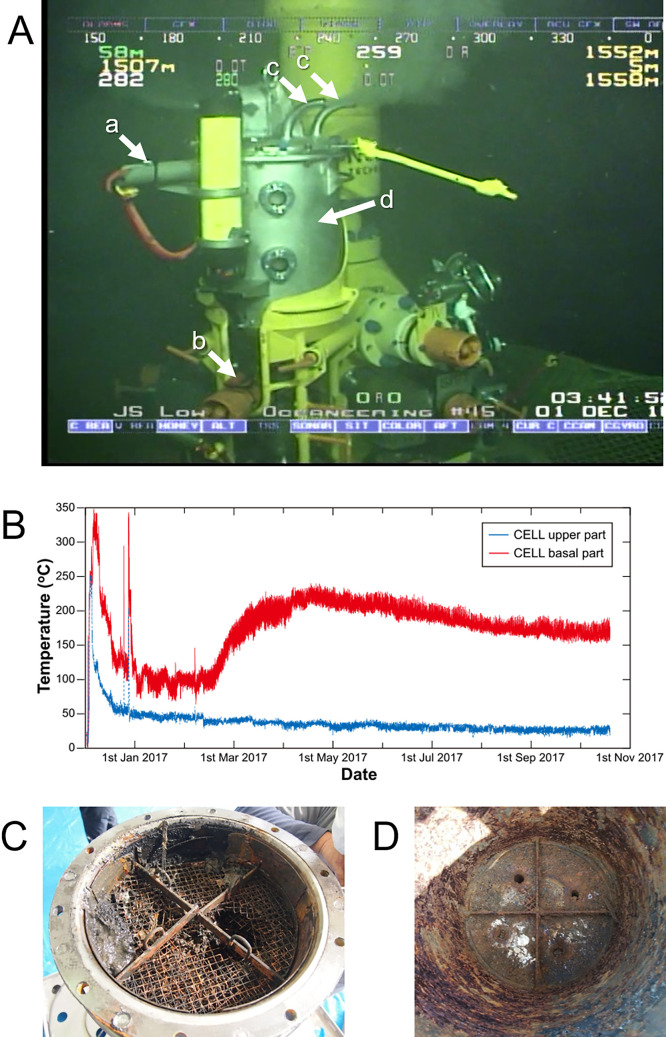
Kuroko-ore cultivation apparatus. (A) Overview of the Kuroko-ore cultivation apparatus. Arrows show two long (a) and short (b) P/T sensors, outlet pipes (c), and the cultivation cell (d). (B) Secular changes in temperature in the upper and basal parts of the cell were monitored during the initial 11 months. (C and D) Pictures of the corroded stainless-steel mesh and the sediment/precipitate that formed in the cell of the Kuroko-ore cultivation apparatus and interior wall.

A geochemical reactor, called the Kuroko-ore (also called black ore, containing hydrothermal sulfide minerals enriched in sphalerite and galena) cultivation apparatus, was installed on the artificial hydrothermal vent at Hole C9017A during the cruise CK16-05. It consisted of an inflow pipe, a cultivation cell, four outlet pipes, and two P/T sensors (Fig. 1A), as previously reported (Nozaki *et al.*, 2021; Kinoshita *et al.*, 2022). Two P/T sensors with short and long probes were inserted into the inflow pipe (basal part of the cell) and the cultivation cell near its top, respectively. Hydrothermal fluid was supplied to the sulfide mineral cultivation cell through the inflow pipe and then flowed out from four outlet pipes on the top of the cultivation cell. After 14.5 months of cultivation, the Kuroko-ore cultivation apparatus was recovered during the cruise KR18-02C, along with the in-side precipitates.

### Sampling

A seal of the Kuroko-ore cultivation apparatus was opened, and six slime-like precipitates were recovered from different positions of the cell (Supplementary Fig. S1). Sample numbers (R01 to R07) corresponded to the order from the top of the cell to the bottom, and sample R06 was only used in another Kuroko-ore cultivation study because of the small amount obtained. Sample R02 was collected from near the center of the cell only, while the other samples consisted of mixtures from near the center and inner walls. These precipitates were recovered into 100-mL medium bottles, and the gas phase of each bottle was replaced with 100% N_2_ gas. These bottles were sealed with a butyl rubber stopper and stored at 4°C until used.

### Microbial cultivation

To enrich iron-corroding microorganisms, 20‍ ‍mL of modified artificial seawater medium supplied with 2.0‍ ‍g of iron granules (Fe >99.98%, 1–2‍ ‍mm in diameter; Alfa Aesar) was prepared using 70-mL serum bottles (Uchiyama *et al.*, 2010). Before the inoculation, the gas phase in the medium bottle was replaced by purging with N_2_/CO_2_ (80:20 ratio) gas. Approximately 1‍ ‍mL of the precipitates recovered from the Kuroko ore cultivation cell was inoculated into the medium, which was then purged again with N_2_/CO_2_ gas. The bottles were sealed with butyl-rubber stoppers and aluminum caps. Bottles containing each precipitate sample were incubated at 37, 50, and 70°C in duplicate.

After the enrichment cultivation, 1‍ ‍mL of the culture was inoculated into 20‍ ‍mL of a newly prepared modified artificial seawater medium containing iron foil (Fe >99.98%, 10×10×0.1‍ ‍mm; Sigma-Aldrich) to conduct the iron corrosion test.

### Analytical method

To evaluate corrosion activity, a 0.5-mL aliquot of the solution containing insoluble matter in the medium was sampled every 7 days after vigorous mixing with a vortex mixer. This was then mixed with 0.5‍ ‍mL of 6 M HCl and 1‍ ‍mL of 1 M L-ascorbic acid. The concentration of iron in the mixture was assessed colorimetrically with orthophenanthroline (Sandell, 1959).

Molecular hydrogen (H_2_) and methane (CH_4_) that accumulated in the headspace of each bottle were measured using a gas chromatograph (GC-3200; GL Sciences) equipped with a thermal conductivity detector and a Molecular sieve 5A column (GL Sciences) at 100°C using N_2_ as a carrier gas.

### DNA extraction

After the enrichment cultivation, 10‍ ‍mL of each culture with insoluble particles of corrosion products and iron particles was sampled, and microbial cells were precipitated by centrifuging at 10,000×*g* for 10‍ ‍min. DNA from the pellet was extracted using a Fast DNA Spin kit for Soil (MP Biomedicals) according to the manufacturer’s protocol with slight modifications. The DNA concentration was measured using a Qubit dsDNA HS assay kit (Thermo Fisher Scientific) and a Qubit 4 Fluorometer (Thermo Fisher Scientific).

### Amplicon sequencing of 16S rRNA gene fragments

Partial 16S rRNA genes (V4–V5 regions) were amplified by PCR with the primer sets 530F and 907R (Caporaso *et al.*, 2012), which contain overhung adapters at the 5' ends. We then performed PCR amplification, enzymatic purification, the addition of multiplexing indices and Illumina sequencing adapters, and purification with magnetic beads as previously reported (Hirai *et al.*, 2017). Amplicon sequencing was conducted using an Illumina MiSeq platform and MiSeq v3 reagent (Illumina) for 300-bp paired-end reads, according to Illumina’s standard protocol.

Raw FASTQ files generated by MiSeq were analyzed using the QIIME2 pipeline (Bolyen *et al.*, 2019). Paired-end FASTQ files were demultiplexed using a demux plugin based on their unique barcodes (Caporaso *et al.*, 2010). The demultiplexed sequences from each sample were treated using the dada2 plugin to obtain the feature table (Callahan *et al.*, 2016). A feature-classifier plugin was then used to align the feature sequences to a pretrained SILVA-138 99% database in order to generate the taxonomy table (Bokulich *et al.*, 2018). Data were rarefied prior to alpha- and beta-diversity ana­lyses using a depth of 40,565 reads. Diversity metrics were calculated and plotted using the core-diversity and emperor plugins, respectively (Vázquez-Baeza *et al.*, 2013). To assess differences among the microbiomes of cultures at each temperature, a permutational multivariate ana­lysis of variance (PERMANOVA) was conducted, which utilized weighted UniFrac distances and was performed with the qiime diversity beta-group significance tool. Each processed file was visualized via QIIME2 View (https://view.qiime2.org), and principal coordinate ana­lysis plot data were exported with the qiime tools.

The datasets generated for this study may be obtained from NCBI using accession codes DRR426559–DRR426587.

## Results

### Corrosion behavior in the Kuroko-ore cultivation apparatus

During cultivation, fluid temperature in the Kuroko-ore cultivation apparatus was monitored in the upper and basal parts of the cultivation cell. The initial temperatures in the upper and basal parts were approximately 250 and 300°C, respectively (Fig. 1B). Fluid temperatures then suddenly decreased to approximately 50 and 100°C, respectively. The temperature in the upper part remained stable, whereas that in the basal part increased again by approximately 200°C, which was maintained for more than 6 months. Unfortunately, temperature measurements ceased in October 2017 because the memory of the P/T sensors reached their capacity limits. After the 14.5-month cultivation, large amounts of precipitates were observed within the cultivation cell, and stainless-steel meshes to trap generated minerals were severely corroded (Fig. 1C). The central part of each stainless-steel mesh had completely dissolved and disappeared. The remaining parts were also corroded, and their color had changed to black or reddish brown. After the removal of the stainless-steel meshes, large amounts of precipitates were observed at the basal part, and the interior wall of the cell had completely corroded (Fig. 1D). Since the dissolved oxygen concentration within the reactor was presumed to be nearly zero, mirroring that of the hydrothermal fluid, corrosion was expected to proceed under anaerobic conditions. Therefore, reddish brown-corrosion products may be formed by oxidization after recovery onboard.

### Enrichment using zero-valent iron particle medium

Six slime-like precipitates containing microbial communities, corrosion products, and generated ore were collected and inoculated into modified artificial seawater medium with zero-valent iron particles. Cultivation was performed at 37, 50, and 70°C, and the dissolved iron concentration of each vial was periodically measured.

**Fig. 2.**
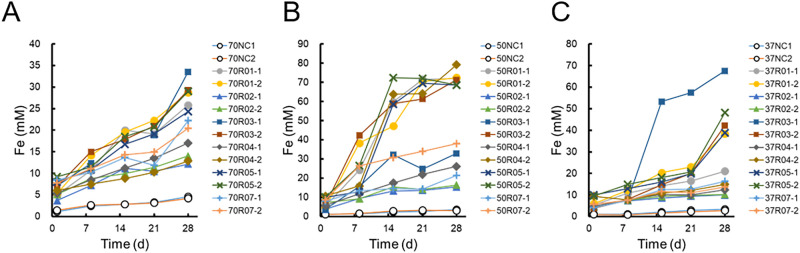
Changes in iron concentrations during the 1st enrichment cultivation at 70°C (A), 50°C (B), and 37°C (C). Open circles represent abiotic controls: 70NC-1, 70NC-2, 50NC-1, 50NC-2, 37NC-1, and 37NC-2.

In the cultivation at 70°C, the dissolved iron concentration of the abiotic control was 4.3‍ ‍mM, while those of the enriched cultures reached 12.1–33.5‍ ‍mM (Fig. 2A). These concentrations increased linearly, and the acceleration ratio against the abiotic control was 2.2- to 8.6-fold higher.

In the cultivation at 50°C, the accelerated dissolution of iron was observed in all cultures (Fig. 2B). The dissolved iron concentration of the abiotic control was 3.3‍ ‍mM, while those of the enriched cultures were 15.4–79.2‍ ‍mM. Therefore, the degree of acceleration was divided into two levels: 1) a 30-fold increase for 50R01-1, 50R01-2, 50R03-2, 50R04-2, 50R05-1, and 50R05-2, and 2) a 4.3–13.7-fold increase for the remaining cultures. In the cultures with higher corrosion activity, the dissolved iron concentration was 2- to 4-fold higher than that in the cultures at 70°C. Dissolution in the 50R01-1, 50R04-2, 50R05-1, and 50R05-2 cultures showed a sigmoid curve, and extremely rapid dissolution was observed (35.6–47.8‍ ‍mM 7 days^–1^).

Since many mesophilic iron-corrosive microorganisms have been reported, we also conducted a cultivation at 37°C. The results obtained showed that the dissolved iron concentration of the abiotic control was 3.0‍ ‍mM, while those of the enriched cultures were 10.0–67.6‍ ‍mM (Fig. 2C). Culture 37R03-1 showed accelerated dissolution during the early cultivation phase, and the dissolution rate reached 45.8‍ ‍mM 7 days^–1^. In contrast, 37R03-2, 37R05-1, and 37R05-2 showed slow dissolution until 21 days and accelerated dissolution (approximately 20‍ ‍mM 7 days^–1^) in the late cultivation phase.

### Iron-corroding test using enriched cultures

**Fig. 3.**
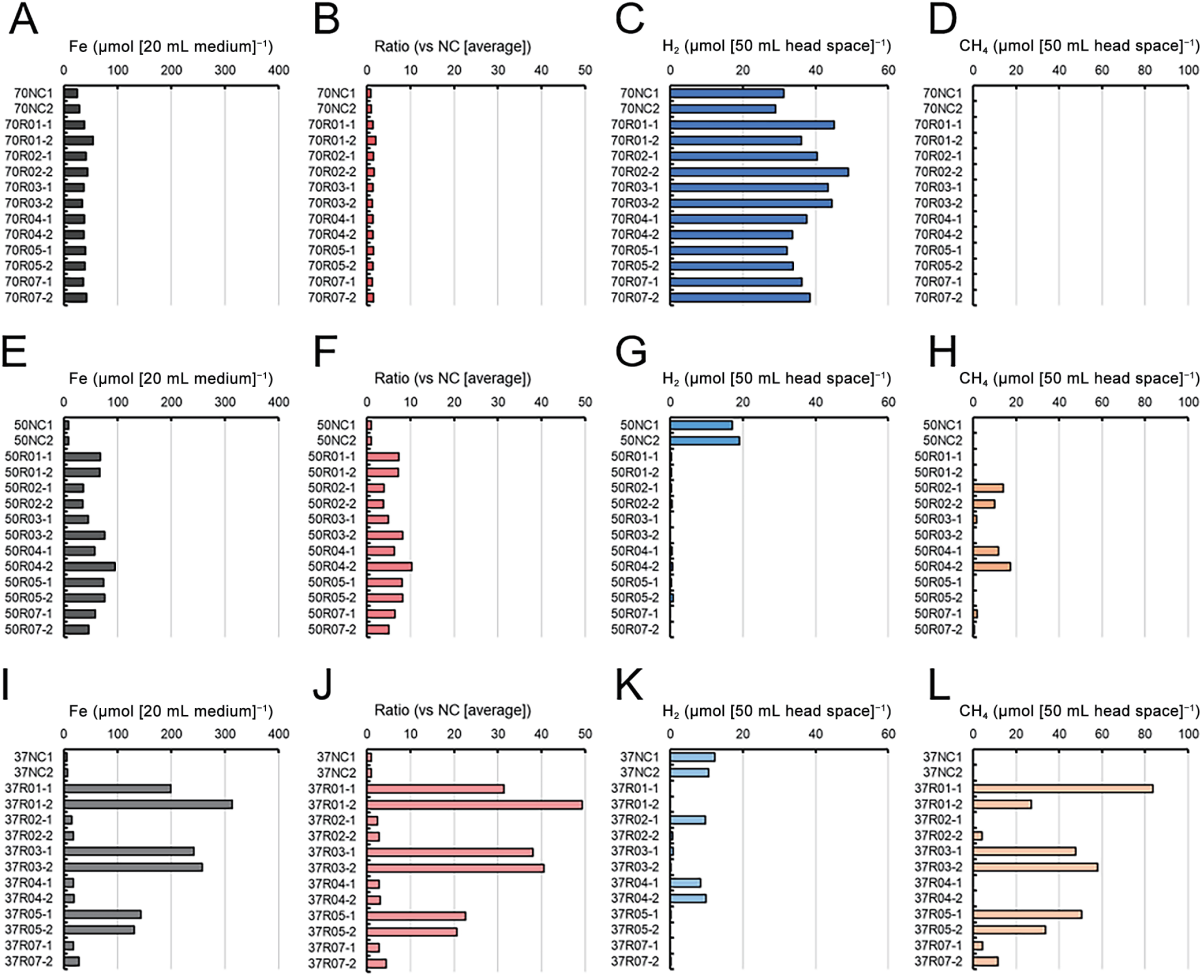
Corrosion test using enriched cultures. Cultivation was conducted at 70°C (A, B, C, and D), 50°C (E, F, G, and H), and 37°C (I, J, K, and L). After cultivation, iron concentrations (A, E, and I) and the amounts of H_2_ (C, G, and K) and CH_4_ (D, H, and L) were measured. Each corrosion acceleration ratio (B, F, and J) was calculated from iron concentrations. NC represents the abiotic control.

To investigate the relationship between corrosion behavior and gas production, a corrosion test was conducted using iron foil and enriched cultures. In the culture at 70°C, the amount of iron dissolution from the iron foil was slightly higher (<2-fold) than that of the abiotic control (Fig. 3A and B). The generation of similar amounts of H_2_ was observed in all cultures containing the abiotic control (Fig. 3C). In contrast, methane was not detected in any of the samples (Fig. 3D).

In the cultures at 50°C, the amount of dissolved iron in all microbial cultures was higher than that in the abiotic control (Fig. 3E), and the ratio of accelerated dissolution was 4- to 10-fold higher (Fig. 3F). Culture 50R04-2 showed the highest ratio, reaching a 10.3-fold increase from the abiotic control. Approximately 20‍ ‍μmol of H_2_ was detected in the abiotic control, whereas only trace amounts were present in the other cultures (Fig. 3G). On the other hand, similar amounts of methane were detected in cultures 50R02-1, 50R02-2, 50R04-1, and 50R04-2 (Fig. 3H).

In the cultures at 37°C, the amount of iron dissolution was clearly divided into two levels: iron dissolution in cultures 37R02-1, 37R02-2, 37R04-1, 37R04-2, 37R07-1, and 37R07-2 was approximately 20‍ ‍μmol, while that in cultures 37R01-1, 37R01-2, 37R03-1, 37R03-2, 37R05-1, and 37R05-2 ranged from 131 to 313‍ ‍μmol (Fig. 3I). The cultures with higher iron dissolution activity showed acceleration ratios that were more than 20-fold higher than that of the abiotic control (Fig. 3J). Notably, culture 37R01-2 had an acceleration ratio that was approximately 50-fold higher. In contrast, cultures with low activity showed only 2- to 5-fold higher acceleration ratios than the abiotic control. Approximately 10‍ ‍μmol of H_2_ was detected in the abiotic control, 37R02-1, 37R04-1, and 37R04-2, while only trace amounts of H_2_ were detected in the other cultures (Fig. 3K). Methane was detected in the cultures with trace amounts of H_2_; notably, culture 37R01-1 had 83.7‍ ‍μmol of CH_4_ (Fig. 3H).

### Relationship between iron dissolution and gas production

The electron equivalent for each reaction was calculated according to the following equations:

Fe^0^→Fe^2+^+2e^–^ (Eq. 1)

2H^+^+2e^–^→H_2_ (Eq. 2)

CO_2_+8H^+^+8e^–^→CH_4_+2H_2_O (Eq. 3).

**Fig. 4.**
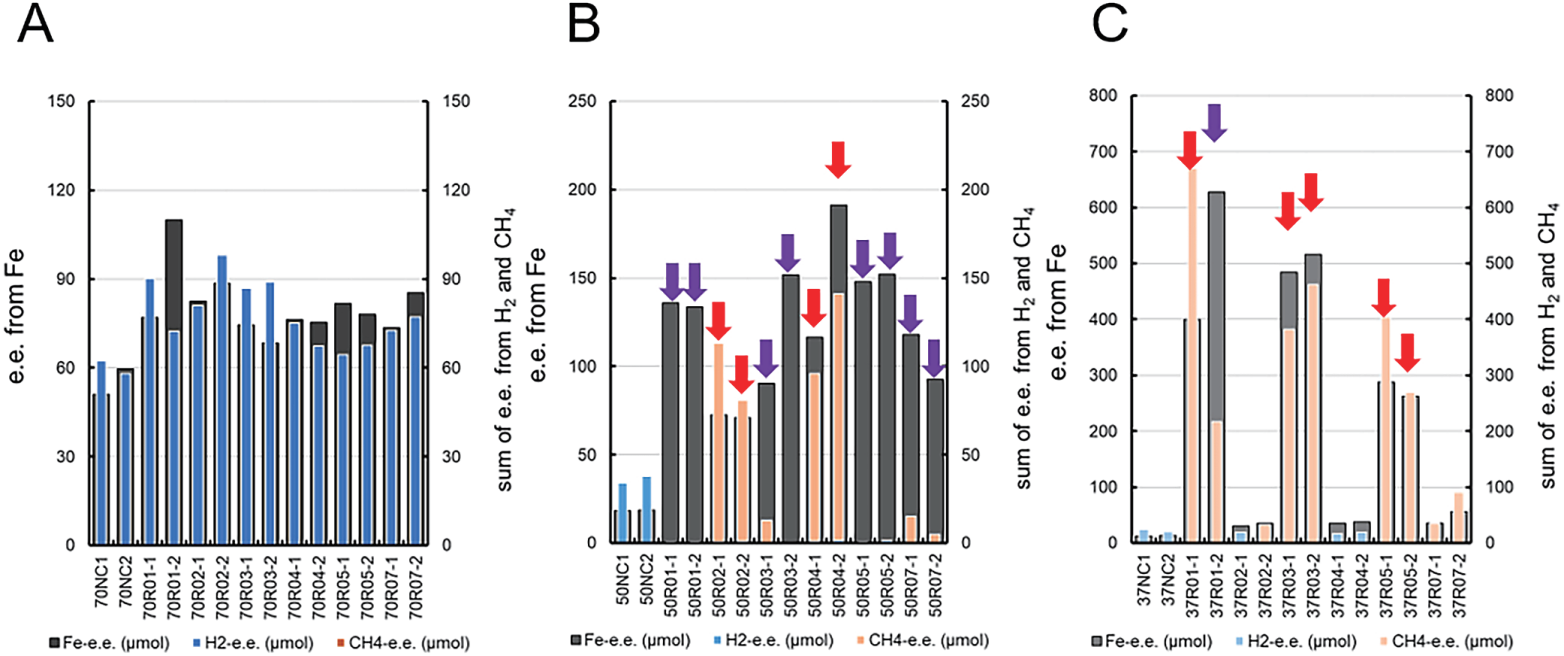
Electron equivalents for iron dissolution and H_2_ and CH_4_ generation at 70°C (A), 50°C (B), and 37°C (C). Purple and red arrows represent H_2_-consumed-type and methanogenesis-type corrosion, respectively. NC represents the abiotic control.

In the cultures at 70°C, each electron equivalent calculated from the amount of iron dissolution and H_2_ generation was similar across all cultures (Fig. 4A). The amount of electrons released from iron foil through iron dissolution corresponded to that consumed during H_2_ generation, according to the following equation:

Fe^0^+2H^+^→Fe^2+^+H_2_ (Eq. 4).

Therefore, these results indicate that corrosion at 70°C was induced by an abiotic chemical reaction.

In the cultures at 50°C, the electron equivalents consumed for methane production were higher than those for abiotic H_2_ generation. Notably, the electron equivalents consumed for methane production in cultures 50R02-1, 50R02-2, 50R04-1, and 50R04-2 were similar to those of iron dissolution (Fig. 4B). In Eq. 1 and Eq. 3, this corrosion reaction may be represented as follows:

4Fe^0^+CO_2_+8H^+^→4Fe^2+^+CH_4_+2H_2_O (Eq. 5).

In contrast, other cultures showed accelerated dissolution without methane production. These results indicated two types of corrosion, namely, methanogenesis-dependent and -independent types.

In the cultures at 37°C, those with higher acceleration ratios showed similar levels of electron equivalents between iron dissolution and methane production, according to Eq. 5; however, these levels in cultures 37R01-1 and 37R01-2 were significantly different (Fig. 4C). The electron equivalent of iron dissolution in culture 37R01-2 was approximately 3-fold higher than that of methane production, indicating a mixture of methanogenesis-dependent and -independent types. In contrast, in cultures 37R02-2, 37R07-1, and 37R07-2, which had low acceleration ratios and weak methane production, the electron equivalents of iron dissolution corresponded to those of methane production. Moreover, in cultures 37R02-1, 37R04-1, and 37R04-2, which had low acceleration ratios and weak H_2_ generation, the electron equivalents of H_2_ generation were similar to those of the abiotic control, while those of iron dissolution were slightly higher.

### Alpha- and beta-diversities of microbial communities in enriched cultures

**Fig. 5.**
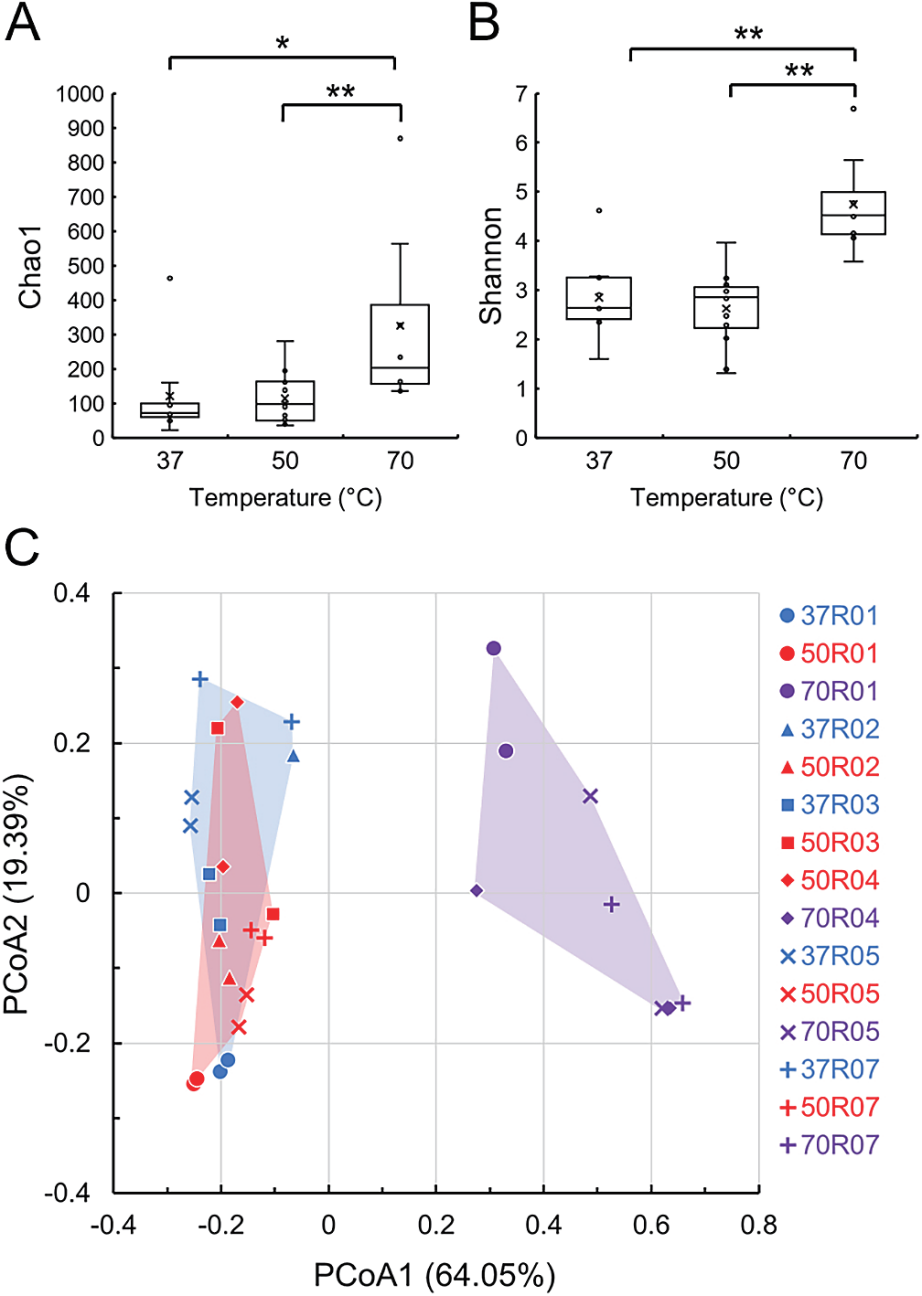
Alpha- and beta-diversities of microbial communities. Chao1 (A) and Shannon (B) indices are represented as box-and-whisker plots. (A) and (B) were calculated based on each cultivation temperature (*n*=9, 12, and 8 for 37, 50, and 70°C, respectively). The line in the middle of the box, top and bottom of the box, whiskers, and cross symbols represent the median, 25 and 75 percentiles, min-to-max values, and average, respectively. Principal coordinate ana­lysis (PCoA) plots based on unweighted UniFrac distances (C). Blue, red, and purple symbols indicate cultures at 37, 50, and 70°C, respectively.

Since differences were observed in accelerated dissolution among the enriched cultures, amplicon sequencing was conducted. Out of 36 enriched cultures, the microbial communities in 29 cultures were analyzed because PCR products were not obtained from the other 7 samples. In the amplicon sequencing ana­lysis, 1,905,643 reads were obtained with a range of 52,187–84,068 reads after denoising and removing chimeric reads (Supplementary Table S1). Using these reads, we calculated Chao1 and Shannon indices as a measure of alpha-diversity. Chao1 and Shannon indices in the enriched cultures at 70°C were 136–869 and 3.58–6.69, respectively, and were higher than those at 50 and 37°C (Fig. 5A and B, and Supplementary Table S1). The principal coordinate ana­lysis plot based on the weighted UniFrac distance showed two clusters of cultures: one at 70°C and another at 37 and 50°C (Fig. 5C). This separation was verified by PERMANOVA (70°C–50°C: *P*=0.001, 70°C–37°C: *P*=0.001, and 50°C–37°C: *P*=0.07). These results suggest that unique microbial communities were formed by enrichment in the cultures at 37 and 50°C.

### Microbial community structures at the phylum level

**Fig. 6.**
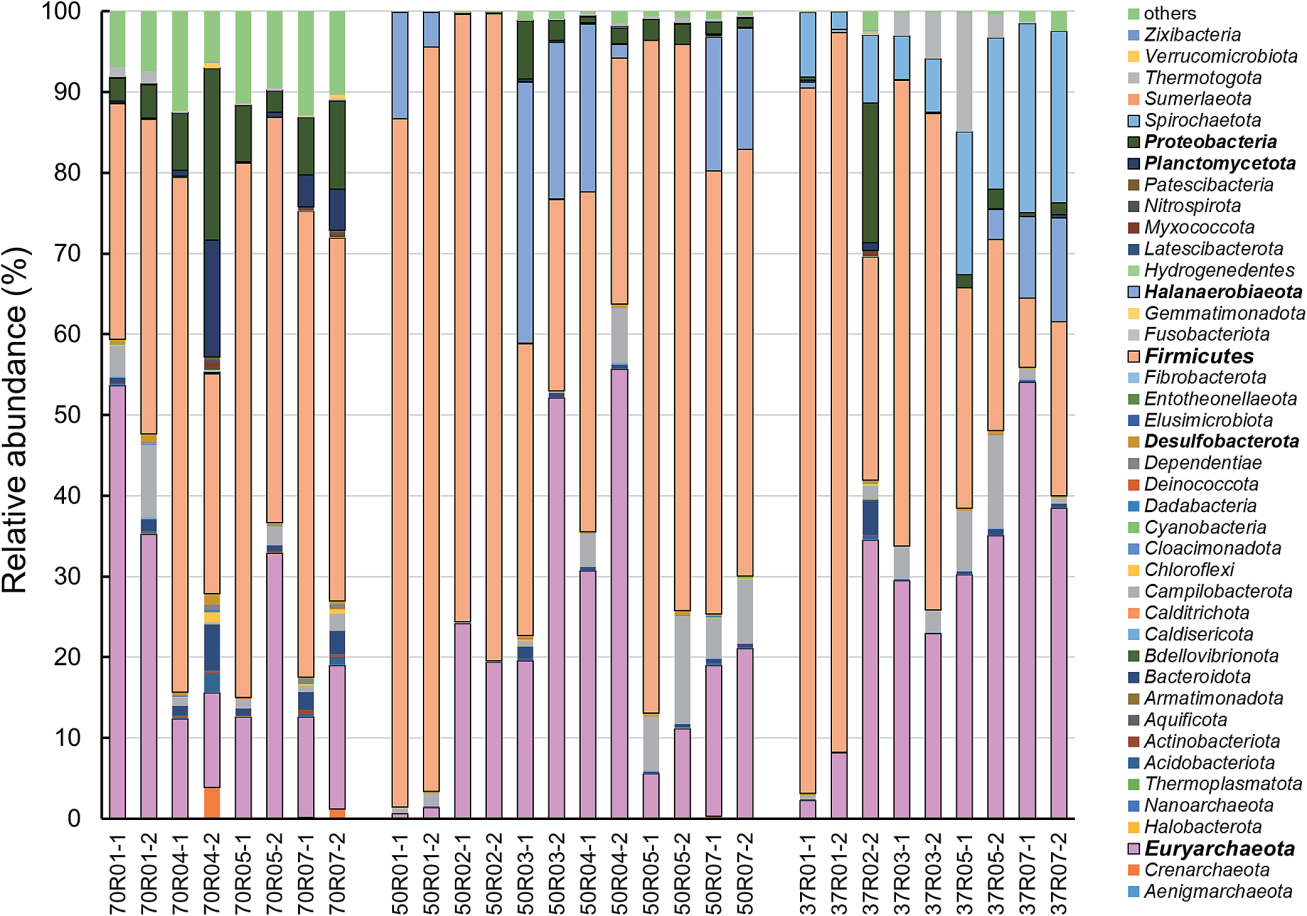
Relative abundance (phylum level) of microorganisms in each sample.

*Firmicutes* and *Euryarchaeota* sequences were observed in the majority of cultures (Fig. 6). In addition, the abundance of *Proteobacteria* and *Planctomycetota* sequences was higher in the culture at 70°C. On the other hand, *Halanaerobiaeota* members were observed in the cultures at 37 and 50°C, while *Spirochaeotota* sequences were only detected in cultures at 37°C. In addition, *Desulfobacterota* members, which are known to contain many SRB and are associated with MIC, showed a minor population of only 0.02–1.32% in amplicon sequence compositions at all temperatures. These results suggest that four major taxa (*Firmicutes*, *Euryarchaeota*, *Proteobacteria*, and *Planctomycetota*) contributed to the accelerated dissolution of iron observed in enrichment cultivation.

### Microbial community structures at the amplicon sequence variant (ASV) level

**Fig. 7.**
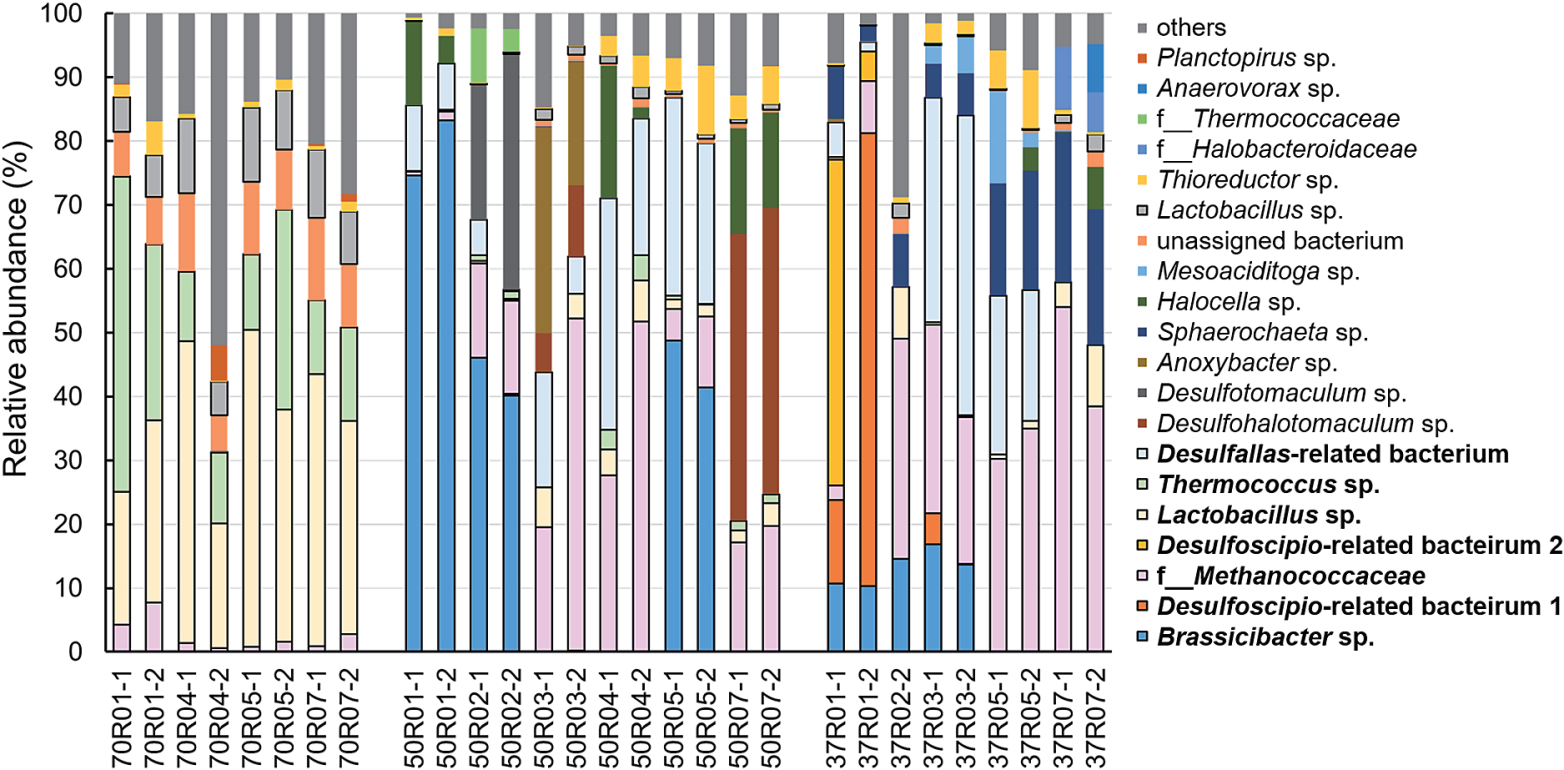
Relative abundance (ASV level) of microorganisms in each sample.

Fig. 7 shows the relative abundance of the top 20 representatives and others at the ASV level. In all cultures, except for 70R04-2 (58.0%), 70R07-1 (79.5%), 70R07-2 (71.7%), and 37R02-2 (71.1%), the sum of these repre­sentatives was higher than 80%. Of the top 20 repre­sentatives, three ASVs were assigned as “genus *Desulfallas-‍Sporotomaculum*”-related bacteria. As a result of the BLAST search using each representative sequence, <97% homology was revealed in the following: “genus *Desulfallas-Sporotomaculum*”, *Desulfoscipio geothermicus* (NR_119245, 96.05%); “genus *Desulfallas-Sporotomaculum* (BRH-c8a)”, *D. geothermicus* (NR_119245, 96.02%); and “genus *Desulfallas-Sporotomaculum* (marine)”, and *Desulfallas thermosapovorans* (NR_119247, 94.96%). Therefore, “genus *Desulfallas-Sporotomaculum*” and “genus *Desulfallas-Sporotomaculum* (BRH-c8a)” were denoted as *Desulfoscipio*-related bacterium 1 and 2, respectively, and “genus *Desulfallas-Sporotomaculum* (marine)” as *Desulfallas*-related bacterium.

In the cultures at 70°C, the sequence of *Lactobacillus* sp. was detected with the highest relative abundance (20.8–49.6%) (Fig. 7). In addition, the sequence of another *Lactobacillus* sp. was observed with 5.5–11.7% relative abundance, and the sum of *Lactobacillus* sp. reached 24.7–61.2%. On the other hand, the sequences of *Thermococcus* sp. (11.0–49.4%), family *Methanococcaceae* (0.6–7.7%), and an unassigned bacterium (5.7–12.3%) were also observed.

In the cultures at 50°C, the sequences of *Brassicibacter* sp., family *Methanococcaceae*, *Desulfohalotomaculum* sp., *Desulfotomaculum* sp., *Desulfallas*-related bacterium, *Anoxybacter* sp., *Halocella* sp., and *Thioreducter* sp. were found in high abundance (Fig. 7). In cultures 50R01-1 and 50R01-2, the percentages of *Brassicibacter* sp. sequences were 74.6 and 83.3%, respectively, while the sequences of *Desulfallas*-related bacterium and *Halocella* sp. were also detected, ranging from 4.3–13.2%. In 50R02-1, 50R02-2, 50R05-1, and 50R05-2, the sequence of *Brassicibacter* sp. had the highest relative abundance (40.2–48.7%). In 50R02-1 and 50R02-2, the *Desulfotomaculum* sp. sequence was the second most abundant (21.1 and 36.8%, respectively), followed by the sequence from the family *Methanococcaceae*, which accounted for 14.8 and 14.6%, respectively. However, in 50R05-1 and 50R05-2, the sequence of *Desulfallas*-related bacterium was the second highest (31.0–25.2%), and the sequences of family *Methanococcaceae* and *Thioreducter* sp. were observed at an abundance of 5.0–11.1%. In 50R03-2 and 50R04-2, the abundance of the family *Methanococcaceae* sequence was the highest (52.0 and 51.7%, respectively). In 50R07-1 and 50R07-2, the sequence of *Desulfohalotomaculum* sp. was observed at the highest percentage (44.9%). Representative microorganisms with the highest abundance in 50R03-1 and 50R04-1 were *Anoxybacter* sp. (32.2%) and the genus *Desulfallas*-related bacterium (36.2%), respectively.

In the cultures at 37°C, the sequences of *Desulfoscipio*-related bacterium 1 and 2, family *Methanococcaceae*, *Desulfallas*-related bacterium, *Brassicibacter* sp., and *Sphaerochaeta* sp. were detected as major representatives (Fig. 7). In 37R01-1 and 37R01-2, *Desulfoscipio*-related bacterium 2 and 1 showed the highest percentages (51.0 and 70.9%, respectively). In other cultures, the sequence of family *Methanococcaceae* was observed at a higher percentage (22.9–54.0%). In addition, in 37R03-1, 37R03-2, 37R05-1, and 37R05-2, the sequence of *Desulfallas*-related bacterium was present at a higher percentage (20.6–47.0%). Moreover, the sequence of *Brassicibacter* sp. was only observed in 37R01-1, 37R01-2, 37R02-2, 37R03-1, and 37R03-2. In contrast, the relative abundance of the *Sphaerochaeta* sp. sequence was lower in these cultures than in the remaining cultures.

## Discussion

In the present study, we investigated microbial populations and their role in MIC by examining corrosion precipitates in a deep-sea hydrothermal environment. Our culture-based corrosion tests confirmed the occurrence of MIC at 50 and 37°C, while no significant MIC was evident at 70°C.

### Microorganisms associated with MIC at 50°C

In the culture-based corrosion test at 50°C, two types of MIC were observed: a methanogenic type and non-methanogenic type. The methanogenic type was detected in cultures 50R02-1, 50R02-2, 50R04-1, and 50R04-2. The electron equivalents consumed for methane production in these cultures were similar to those used for iron dissolution, as indicated by Eq. 5. However, these equivalents were 2- to 4-fold higher than those consumed for H_2_ generation in the abiotic control (Fig. 4). Accelerated corrosion cannot be explained by only methane production based on the consumption of H_2_ resulting from chemical corrosion, as denoted in Eq. 4. Therefore, we classified accelerated corrosion as methanogenic-type MIC. Methanogenic metabolism appears to directly correlate with the accelerated dissolution of iron. It is important to note that MIC by some methanogens has already been reported (Dinh *et al.*, 2004; Mori *et al.*, 2010; Uchiyama *et al.*, 2010; Hirano *et al.*, 2022).

**Fig. 8.**
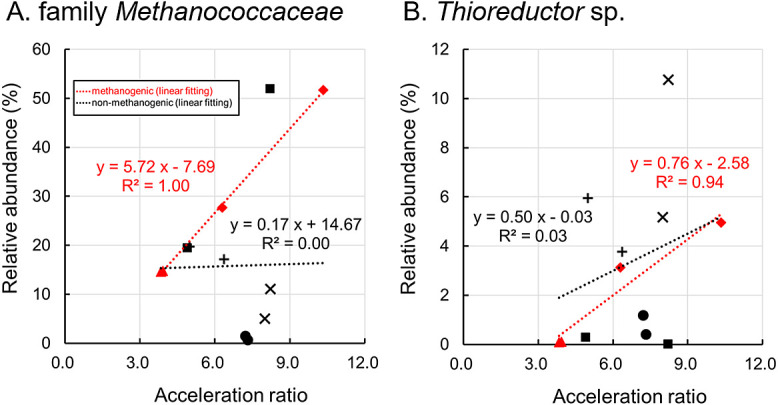
Relationship between the acceleration ratio and relative abundance of family *Methanococcaceae* (A) and *Thioreductor* sp. (B) in the cultivation at 50°C. Red and black symbols represent methanogenic and non-methanogenic types, respectively. Circle, triangle, square, rhombus, cross, and plus represent samples R01, R02, R03, R04, R05, and R07, respectively.

Although family *Methanococcaceae* sequences were observed in almost all cultures at 50°C, the acceleration ratios in methanogenesis-dependent corrosion positively correlated with the relative abundance of the archaeal sequences (Fig. 8A). A BLAST search using the representative‍ ‍se­quence of the family *Methanococcaceae* observed in these cultures revealed the highest identity with the thermo­philic,‍ ‍hydrogenotrophic methanogen, *Methanothermococcus okinawensis* strain IH-1 (accession number NR_102915, 100% identity). Since a culture strain of *M. okinawensis* was isolated from the hydrothermal chimney in the same deep-sea hydrothermal field (Takai *et al.*, 2002), the detection of this archaeon appears to be reasonable. Although MIC by the genera *Methanobacterium* and *Methanococcus* has been reported (Dinh *et al.*, 2004; Mori *et al.*, 2010, Uchiyama *et al.*, 2010, Hirano *et al.*, 2022), there is currently no information on MIC by the genus *Methanothermococcus*.

In addition to the family *Methanococcaceae*-related archaeon, a positive correlation was observed between the percentage of *Thioreductor* sp. sequences and acceleration ratios (Fig. 8B). The BLAST search, using the representative sequence of *Thioreductor* sp., showed the highest identity with the mesophilic, sulfate-reducing bacterium, *Thioreductor micantisoli* BKB25Ts-Y (accession number NR_041022, 98.11% identity). Although *T. micantisoli* BKB25Ts-Y was isolated from sediment near a hydrothermal vent in the same deep-sea field (Nakagawa *et al.*, 2005), this strain cannot grow at 50°C, which was the temperature in the enrichment and corrosion test. Therefore, the *Thioreductor* sequence observed in the culture may be a moderately thermophilic bacterium related to *T. micantisoli*. Similar to the family *Methanococcaceae*-related bacterium, there is currently no information on MIC by the genus *Thioreductor*.

The sequences of family *Methanococcaceae* and *Thioreductor* sp. were detected in the majority of cultures at 50°C. Additionally, a positive correlation was observed between the percentages of these sequences and accel­eration‍ ‍ratios in methane-producing cultures. Therefore, methanogenic-type corrosion may result from the cooperative activity of methanogens and SRB, classifying them to‍ ‍methanogen–sulfate-reducing bacteria cooperative-type‍ ‍corrosion. In support of this result, corrosion by *Methanococcus maripaludis* KA1 was enhanced by a co-cultivation with SRB (Wakai, 2020). According to the proposed model, coexisting SRB have the capacity to generate hydrogen sulfide, which subsequently reacts with iron carbonate, a corrosion byproduct in MIC facilitated by iron-corrosive methanogens. This reaction generates electro-conductive iron sulfide and releases carbonate ions. Iron-corrosive methanogens then utilize dissolved inorganic carbon released from the inert corrosion product as their methanogenic substrate. Iron-corrosive *M. maripaludis* KA1‍ ‍and OS7 have an extracellular hydrogenase, which is‍ ‍homologous to the hydrogenase from the genus *Methanobacterium* and, thus, accelerated corrosion by these methanogens is considered to be the EMIC type based on the extracellular hydrogenase (Tsurumaru *et al.*, 2018). Therefore, the family *Methanococcaceae* member enriched in the cultivation test may also possess a similar corrosion mechanism. Alternatively, corrosion through electrosyntrophic metabolism has been proposed. Based on previous findings showing electrosyntrophic growth in micro­organisms with electrochemical activity (Kato *et al.*, 2012), the co-existence of the family *Methanococcaceae* and *Thioreductor* sp. observed in the enriched cultures may be mutually beneficial via electrosyntrophic energy metabolism during the corrosion process.

Non-methanogenic corrosion was also observed. In these cultures, the percentage of specific representative microorganisms did not correlate with acceleration ratios (Supplementary Fig. S2A). In cultures 50R01-1 and 50R01-2, *Brassicibacter* sp. was significantly enriched (Fig. 7). However, the presence of this bacterium may not directly correlate with the corrosion process based on our BLAST search results. This search revealed that *Brassicibacter* sp. is closely related to the mesophilic heterotroph, *Brassicibacter mesophilus* (accession number NR_10884, 99.73%), which does not possess the ability to utilize reduced sulfur compounds or known EET systems (Fang *et al.*, 2012). In addition, the sequence of *Brassicibacter* sp. was detected at a relatively high abundance in cultures 50R02-1, 50R02-2, 50R05-1, and 50R05-2, among which 50R02-1 and 50R02-2 are methanogenic-type MIC. These results indicate that *Brassicibacter* sp. is irrelevant to the acceleration of corrosion. On the other hand, SRB, such as *Desulfallas*-related bacterium, *Desulfohalotomaculum* sp., and *Thioreductor* sp., were detected in non-methanogenic-type corrosion (Fig. 7). Therefore, these SRB may accelerate corrosion through the CMIC or EMIC system.

### Microorganisms associated with MIC at 37°C

**Fig. 9.**
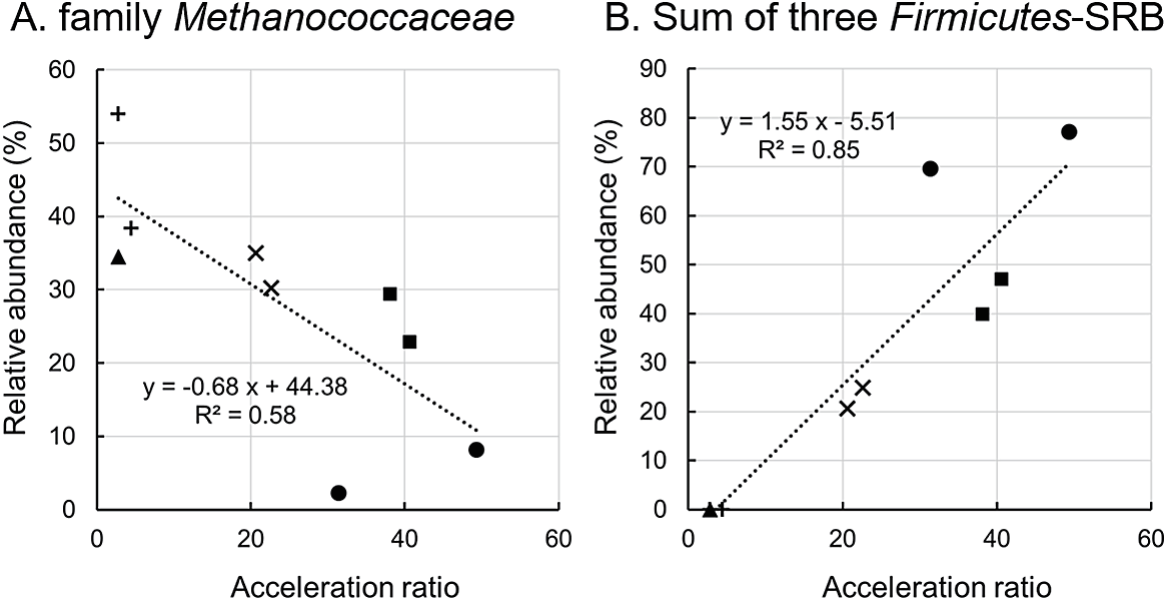
Relationship between the acceleration ratio and relative abundance of family *Methanococcaceae* (A) and the sum of three *Firmicutes*-SRB (B) in the cultivation at 37°C. Circle, triangle, square, cross, and plus represent samples R01, R02, R03, R05, and R07, respectively.

Six cultures showed highly accelerated methanogenic-type corrosion: 37R01-1, 37R01-2, 37R03-1, 37R03-2, 37R05-1, and 37R05-2 (Fig. 3 and 4). However, the abundance of family *Methanococcaceae* sequences negatively correlated with the acceleration ratio (Fig. 9A). Therefore, methanogenic energy metabolism may have contributed to some of the corrosion process, while other forms of microbial metabolism may have enhanced this process. In culture 37R01-2, which exhibited low methane production relative to the acceleration ratio, the sequence of *Desulfoscipio*-related bacterium 1 accounted for 70.9% of the microbial community’s composition (Fig. 7). In addition, the sequences of other SRB, such as *Desulfoscipio*-related bacterium 2 and *Desulfallas*-related bacterium, were observed at higher percentages in the cultures with high acceleration ratios (Fig. 7). Although these SRB and other abundant microorganisms did not show a positive correlation with the acceleration ratio (Supplementary Fig. S2B), the sum of the percentages of these three SRB positively correlated with the acceleration ratio (Fig. 9B). Therefore, these SRB and members of family *Methanococcaceae* may be key players and electron consumers, respectively, in accelerated corrosion at 37°C.

The aforementioned SRB belong to the phylum *Firmicutes*, whereas the representative SRB related to MIC in many previous studies are members of the phylum *Desulfobacterota*, including the genus *Desulfovibrio* (Enning *et al.*, 2012; Waite *et al.*, 2020). Of these, *Desulfovibrio ferrophilus* and *Desulfopila corrodens* were shown to significantly accelerate corrosion through EMIC via EET (Dinh *et al.*, 2004; Enning *et al.*, 2012; Enning and Garrelfs, 2014; Kato *et al.*, 2016), and multi-heme-type outer membrane cytochromes played an important role. In addition, MIC by *Desulfovibrio vulgaris* has been demonstrated, and this microorganism has also been shown to induce CMIC-type corrosion because it lacks the outer membrane cytochrome required for EET. The relative species of *Firmicutes*-SRB enriched in our cultures included *D. geothermicus* (accession number NR_119245, 96.0% identity) and *D. thermosapovorans* (accession number NR_119247, 94.96% identity). Although their genome sequences have been analyzed, homologues of the outer membrane cytochrome have yet to be annotated, indicating that *Firmicutes*-SRB may not participate in EMIC-type corrosion. However, the acceleration ratios in these cultures were 20- to 49.6-fold higher than those of the abiotic control, and this significantly higher corrosive activity is difficult to explain through the corrosion mechanism of CMIC. Since biogenic iron sulfide nanoparticles have been shown to enhance EET (Deng *et al.*, 2020), in *Firmicutes*-SRB enriched cultures, these microorganisms may acquire EET through a similar mechanism, subsequently accelerating corrosion and, thus, we classified them to sulfate-reducing *Firmicutes*-induced-type corrosion.

## Conclusion

In the present study, we provided evidence for MIC caused by moderately thermophilic and mesophilic microorganisms from deep-sea environments. In addition, we found that methanogenic-type corrosion predominated at 50°C, while *Firmicutes*-SRB significantly accelerated corrosion at 37°C. While a quantitative assessment of the contribution of these microorganisms to corrosion is required, these microorganisms appear to play an important role in MIC in natural environments. However, previous studies on MIC primarily focused on sulfur-metabolizing bacteria in cold deep-sea environments. The present results provide insights into MIC in warm and hot deep-sea environments, bridging the gap in the literature and providing valuable insights for future research in this field.

## Citation

Wakai, S., Sakai, S., Nozaki, T., Watanabe, M., and Takai, K. (2024) Accelerated Iron Corrosion by Microbial Consortia Enriched from Slime-like Precipitates from a Corroded Metal Apparatus Deployed in a Deep-sea Hydrothermal System. *Microbes Environ ***39**: ME23089.

https://doi.org/10.1264/jsme2.ME23089

## Supplementary Material

Supplementary Material
