# Supplementary material for: Accelerated Iron Corrosion by Microbial Consortia Enriched from Slime-like Precipitates from a Corroded Metal Apparatus Deployed in a Deep-sea Hydrothermal System
Source: Microbes Environ. 2024 Jun 6;39(5):ME23089. doi: 10.1264/jsme2.ME23089 (PMC11946385; doi:10.1264/jsme2.ME23089)
Supplement: Supplementary file 1 — Supplementary Material [file 39_23089_s1.pdf]

Supplementary Material

Accelerated Iron Corrosion by Microbial Consortia Enriched from Slime-like Precipitates from a Corroded Metal Apparatus Deployed in a Deep-sea Hydrothermal System

Satoshi WAKAI[1,2*], Sanae SAKAI[1], Tatsuo NOZAKI[3,4,5],

Masayuki WATANABE[3], and Ken TAKAI[1]

[1] Institute for Extra-cutting-edge Science and Technology Avant-garde Research (X-star), Japan Agency for Marine-Earth Science and Technology (JAMSTEC), Yokosuka, Japan; [2] PRESTO, Japan Science and Technology Agency (JST), Tokyo, Japan; [3] Submarine Resources Research Center, Research Institute for Marine Resources Utilization, Japan Agency for Marine-Earth Science and Technology (JAMSTEC), Yokosuka, Japan; [4] Frontier Research Center for Energy and Resources, School of Engineering, The University of Tokyo, Japan; [5] Department of Planetology, Graduate School of Science, Kobe University, Kobe, Japan

Correspondence and requests for materials should be addressed to S.W. (Tel.: +81-46-867-9693, e-mail: wakais@jamstec.go.jp)

Table S1 Sequencing features and alpha diversities

Samples	Input	Non-chimeric	Observed features	Chao1	Shannon entropy
70R01-1	142,069	79,274	163	163	3.58
70R01-2	101,135	54,439	171	171	4.50
70R04-1	118,044	61,457	234	234	4.54
70R04-2	136,361	69,392	866	869	6.69
70R05-1	111,306	55,278	136	136	4.15
70R05-2	103,515	52,187	136	136	4.06
70R07-1	110,302	57,635	327	327	4.78
70R07-2	127,312	64,576	562	563	5.65
50R01-1	123,425	60,867	39	39	1.39
50R01-2	126,473	69,027	52	52	1.31
50R02-1	137,854	63,363	41	41	2.29
50R02-2	137,279	66,253	33	35	2.02
50R03-1	128,213	71,611	278	281	3.96
50R03-2	123,741	73,366	166	167	2.83
50R04-1	134,092	80,230	138	138	2.87
50R04-2	100,131	58,305	106	106	2.98
50R05-1	118,644	61,423	65	65	2.48
50R05-2	127,201	66,112	90	90	3.05
50R07-1	122,083	65,805	194	194	3.24
50R07-2	113,718	64,718	161	162	3.11
37R01-1	143,381	64,202	98	99	2.62
37R01-2	128,161	58,567	21	21	1.60
37R02-2	151,758	84,068	459	463	4.62
37R03-1	119,987	67,484	59	59	2.63
37R03-2	108,496	61,432	50	50	2.35
37R05-1	107,753	63,642	71	71	2.91
37R05-2	114,363	67,922	94	95	3.26
37R07-1	114,492	71,380	68	69	2.41
37R07-2	118,656	71,628	159	159	3.28

Supplementary Figures

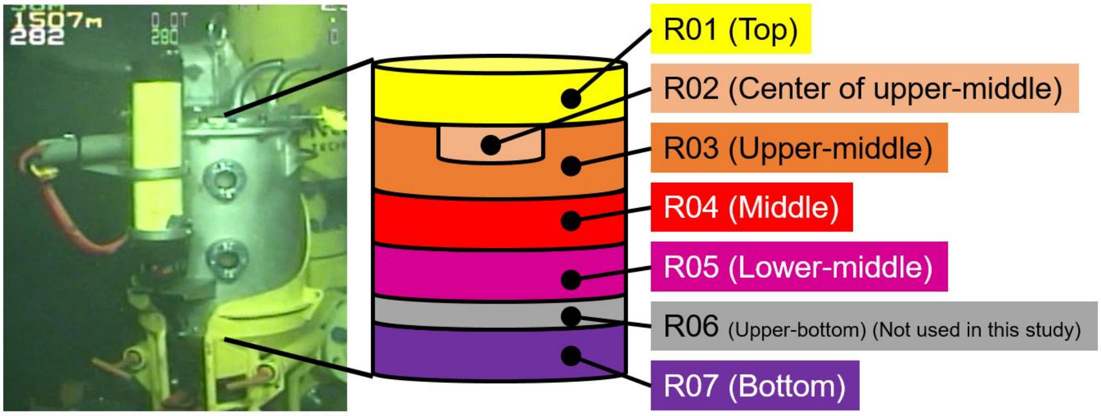

Fig. S1. The positions of sampled slime-like precipitates in the cell of Kuroko-ore cultivation apparatus.

A. Corrosion test at 50°C

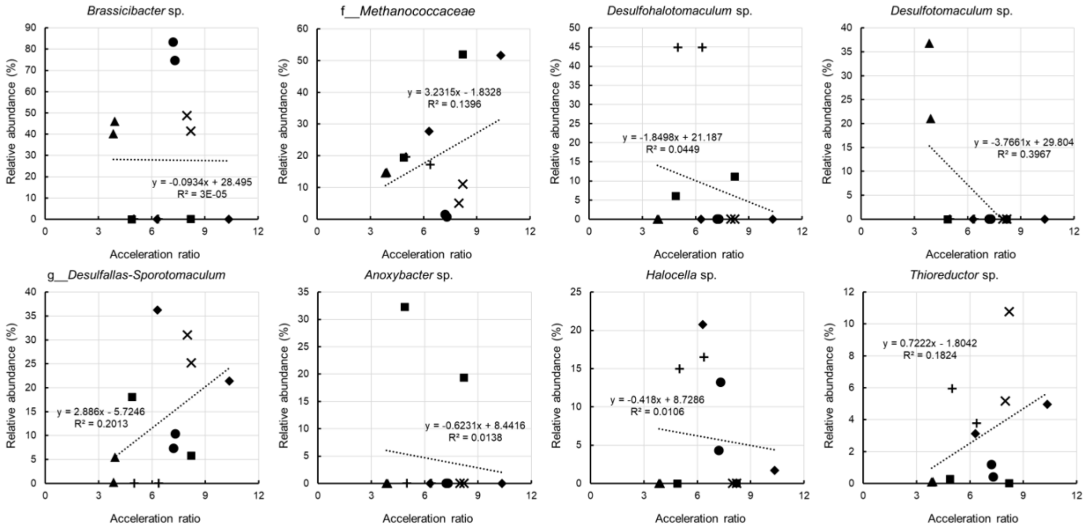

B. Corrosion test at 37°C

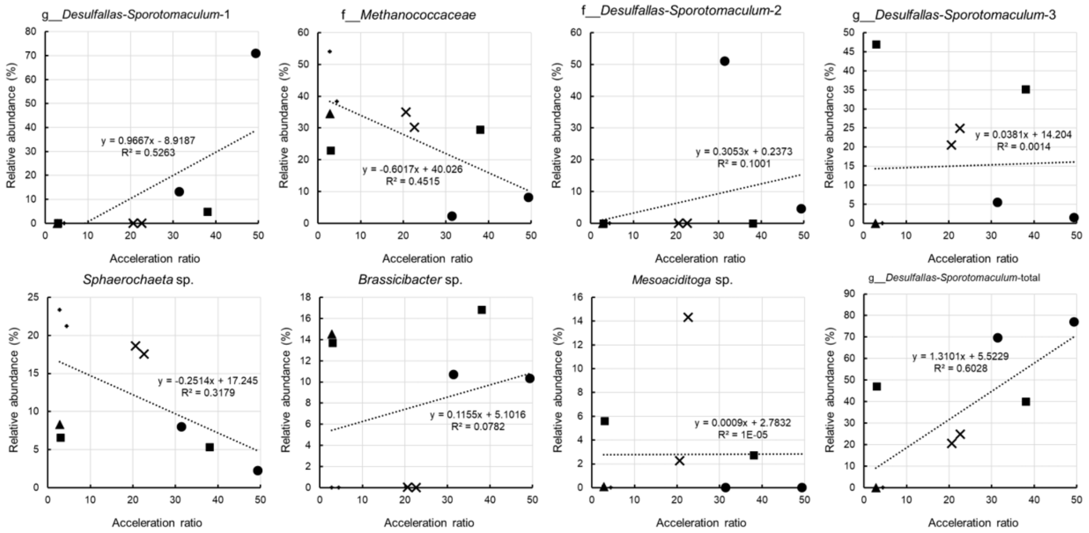

Fig. S2. Correlation between the acceleration ratio and relative abundance of each ASV in the cultivation at 50°C (A) and 37°C (B). Circle, triangle, square, rhombus, cross, and plus represent the samples R01, R02, R03, R04, R05, and R07, respectively.
